# Effect of the Advanced Cranial and Craniofacial Implant Fabrication on Their Degradation Affinity

**DOI:** 10.3390/ma16176070

**Published:** 2023-09-04

**Authors:** Edyta Chmal-Fudali, Daria Basińska, Agnieszka Kucharska-Jastrząbek, Marcin H. Struszczyk, Małgorzata Muzalewska, Marek Wyleżoł, Marcin Wątrobiński, Jacek Andrzejewski, Nina Tarzyńska, Karolina Gzyra-Jagieła

**Affiliations:** 1 Institute of Security Technologies “MORATEX”, 3 M. Sklodowskiej-Curie Str., 90-505 Lodz, Poland; efudali@moratex.eu (E.C.-F.); dbasinska@moratex.eu (D.B.); ajastrzabek@moratex.eu (A.K.-J.); 2 Department of Fundamentals of Machinery Design, Faculty of Mechanical Engineering, Silesian University of Technology, Konarskiego 18a Str., 44-100 Gliwice, Poland; malgorzata.muzalewska@polsl.pl (M.M.); marek.wylezol@polsl.pl (M.W.); 3 Syntplant, Rubież 46/C4, 61-612 Poznan, Poland; office@syntplant.com; 4 Institute of Materials Technology, Poznan University of Technology, Piotrowo 3 Str., 61-138 Poznan, Poland; jacek.andrzejewski@put.poznan.pl; 5 Lukasiewicz Research Network—Lodz Institute of Technology, 19/27 M. Sklodowskiej-Curie Str., 90-570 Lodz, Poland; nina.tarzynska@lit.lukasziewicz.gov.pl (N.T.); karolina.gzyra-jagiela@lit.lukasiewicz.gov.pl (K.G.-J.)

**Keywords:** biodegradable cranial, craniofacial implants, biodegradation in vitro test, 3D printing, injection, PLA, nanohydroxyapatite

## Abstract

Biodegradable craniofacial and cranial implants are a new aspect in terms of reducing potential complications, especially in the long term after surgery. They are also an important contribution in the field of surgical reconstructions for children, for whom it is important to restore natural bone in a relatively short time, due to the continuous growth of bones. The aim of this study was to verify the impact of the technology on biodegradability and to estimate the risk of inappropriate implant resorption time, which is an important aspect necessary to select prototypes of implants for in vivo testing. Prototypes of implants were made using two technologies: 3D printing using a PLDLA: poly(L-co-D,L lactide) (PLDLA) filament containing hydroxyapatite nanoparticles, and injection using PLDLA. After the radiation sterilization process, they were subjected to in vitro degradation under accelerated conditions. As part of this study, the in vitro degradation of newly developed biodegradable implant technologies was assessed in accordance with the guidelines of European standards. It was found that the implant manufacturing process had a significant impact on the degradation time under simulated conditions in various media. Implants made using the injection technique were characterized by lower susceptibility to degradation media compared to the 3D-printed implant under accelerated conditions.

## 1. Introduction

The dynamic development of technologies of advanced, biodegradable medical devices mainly results from the aging of the population, the increase in the number of injuries resulting from accidents, the frequency of acquired or congenital facial deformities, and the need to ensure the safest and most effective medical procedures, ensuring a high level of aesthetics of the reconstructions performed [1]. A special group of patients for whom biodegradable craniofacial and/or cranial implants are an important aspect are people under 18 years of age, for whom the development process has not been completed. In these cases, the use of typical, non-resorbable implants is not possible, due to the need to replace them during the growth and development of the child.

The application of 3D printing allows us to customize the implant’s shape for optimal fitting to the bone defect as well as to personalize the bioactive additive content and the porosity for optimal healing. A flexibility in the additive amount with the increase in the mechanical properties of the resulting implants is achieved through the implementation of ceramic-based compounds in polycaprolactone via binder jetting conjugated with capillary rise infiltration [2]. Moreover, 3D printing allows, due to its technical flexibility, the addition of several bioactive substances originating from biominerals: eggshell, pearl, turtle shell, degelatinated deer antler, and cuttlebone [3].

Zimmermann et al. [4] estimated the degradation of a biodegradable composite with a polylactide acid (PLA) matrix containing spherical magnesium microparticles. The degree of degradation was assessed through hydrogen release from the studied materials. In study [5], the mineral-PLLA composites fabricated using injection were subjected to degradation in vitro in Hank’s balanced salts solution. The pH and the polarization of the degradation media were controlled. Micro-computed tomography was applied for the assessment of the degradation yield of biodegradable implants to describe the degradation homogeneity in 3D [6]. The process of hydrolytic degradation of PLGA untreated or treated by a CO_2_ laser carried out in distilled water is described in [7]. The PBS buffer medium was used for the estimation of the degradation affinity of the PLA-PCL fibers at a temperature of 37 °C [8], 3D bioabsorbable PLA fabric scaffolds at 58 °C [9], and cylinder samples made through the 3D printing of mixtures of polylactic acid and polyhydroxy butyrate [10]. A similar medium at various pH was applied to assess the degradation profile of the PEOT-PBT and poly(ester urethane) films [11]. Bremrer et al. [12] used the guide of [13] for the evaluation of the degradation in vitro potentiality of the poly(ester)urethane-based adhesive. The simulated body fluid (SBF) at 37 °C was also used for the assessment of the degradation profile of the bi-layered 3D-printed scaffold with the PLA layer and PLA/bioglass G5 layer [14]. 

The profile of the in vitro degradation of the various usable forms and compositions containing PLA differed in relation to the content and proportion of the additives [4,5,6,7,8,9,10,11,12,13,14]. Bogdanova et al. [15] received the 10% weight loss only during the in vitro degradation of the electrospun PLA-gelatin composition for 8 weeks in PBS and Fenton’s solution. Degradation of the PLLA stents carried out in PBS medium at 70 °C resulted in the disintegration of the stent structure after 12 days [16]. On the other hand, the application of PBS media at 50 °C for the in vitro degradation prolonged the degradation up to 16 weeks without substantial weight loss of the PLLA stents [17].

PLA degrades in the patient’s body, initially through hydrolysis of ester bonds, resulting in a mixture of lactic acid oligomers and its monomers. The process of hydrolytic degradation is accelerated by the formation of carboxyl groups, causing a decrease in the pH of the environment [18]. The possibility of diffusion of water molecules into the interior of an implant made of PLA causes the destruction of the implant structure by the pawing of spaces filled with PLA hydrolysis products and with a reduced pH in relation to the external surface [19]. Another factor supporting the degradation process of PLA is temperature. The higher it is, the faster the PLA degradation process occurs [20,21]. For the above reason, when designing studies on the degradation profile of medical devices made of PLA, these two factors, important for the rationality of the obtained test results, should be taken into account.

The introduction of a biodegradable medical device is conditional on conducting tests aimed at estimating the biodegradation profile, so that before animal tests, there are data enabling the assessment of the assumptions made regarding the resorption time. This reduces the risk of non-compliant prototypes being subjected to in vivo testing (validation process) and does not compromise animal welfare. As indicated in [12], in vitro testing is indicated by regulatory authorities as indispensable to reduce the number of negative tests on animals. However, the results of these in vitro tests may not be sufficient to confirm the safety and performance of the medical device, as they are only a simplified research model. Nevertheless, they give basic answers in terms of the correctness of estimating the assumed biodegradation period, which should be confirmed under in vitro conditions. 

The aim of this study was to estimate the degradation time in vitro of two prototypes of implants fabricated by 3D printing from a PLDLA filament with hydroxyapatite nanoparticles or injection from PLDLA without hydroxyapatite and to assess the impact of the technology on the degradation time in various media. In this research thesis, it was established that the choice of implant technology will significantly affect the degradation time under simulated conditions.

## 2. Materials and Methods

### 2.1. Materials

The polylactide type used during this study was commercially available PLDLA co-polymer Purasorb PLDL 8058 (Corbion/Amsterdam, The Netherlands). This type of medical-grade copolymer is characterized with an inherent viscosity of 5.8 dL/g, and the L-lactide/DL-lactide molar ratio was 80/20. Hydroxyapatite (HAp) filler in the form of white powder was supplied by PPNT (Poznan/Poland).

Two types of implant prototypes were used: (a) that fabricated through 3D printing using filament with PLDLA copolymer containing 10 wt% hydroxyapatite particles, and (b) that fabricated through injection using PLDLA copolymer. 

For the degradation test, the following materials were used: deionized water, hydrogen peroxide (Chempur/Piekary Śląskie, Poland), NaCl (Chempur/Poland), NaHCO_3_ (Chempur/Poland), KCl (Chempur/Poland), K_2_HPO_4_ × 3H_2_O (Chempur/Poland), MgCl_2_ × 6H_2_O (Chempur/Poland), HCl (1 M; Chempur/Poland), CaCl_2_ (Chempur/Poland), Na_2_SO_4_ (Chempur/Poland), indium (Chempur/Poland), and n-octane (Chempur/Poland).

#### Preparation of Artificial Saline

To prepare 1000 mL of artificial plasma, 700 cm^3^ of deionized water was placed in a plastic beaker and heated to 36.5 ± 1.5 °C under stirring with a magnetic stirrer (type 6MM; Polamed/Warszawa, Poland). Then, the following reagents were added under stirring: KCl (8.035 g), NaHCO_3_ (0.355 g), KCl (0.225 g), K_2_HPO_4_ × 3H_2_O (0.231 g), MgCl_2_ × 6H_2_O (0.311 g), HCl (39 cm^3^), CaCl_2_ (0.292 g), and Na_2_SO_4_ (0.072 g). The pH of the prepared medium ranged from 7.42 to 7.45. The fabricated medium was filled up to 1000 cm^3^ with deionized water. 

### 2.2. Methods

#### 2.2.1. Fabrication of the Implants’ Prototypes

##### 3D Printing

**Figure 1:**
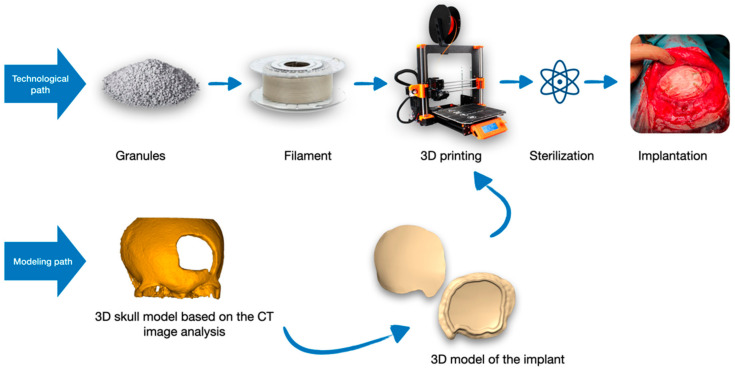
General diagram of the implant fabrication process via 3D printing.

The process of manufacturing an implant using 3D printing starts with two paths: the first one (technological path) relates to filament production technology, and the second one (modeling path) involves modeling the implant based on a 3D model of the anatomical structure obtained through medical imaging analysis (Figure 1). Both paths converge in the 3D printer, where, based on the virtual model and the produced filament, the implant is printed. 

The printed implant undergoes sterilization by accelerated electrons and is then ready for implantation.

The 3D printing procedure consists of filament fabrication and the main 3D printing process. The filament preparation stage was carried out on a METALCHEM W25-30D single-screw machine (IMPiB/Toruń, Poland). The maximum preset extrusion temperature of the head was 210 °C, while the rotational speed of the screw was set at 10 rpm. The diameter of the extruder nozzle was 2 mm, while the final fiber diameter of about 1.75 mm was controlled by the take-off speed, which was 5 to 7 m/min depending on the melt viscosity. The filament was cooled in a stream of cold air, and the finished material was wound onto spools using a winder. A more detailed description of the material fabrication procedure is provided in our previous research [22].

The 3D printing process was carried out on a Prusa MK3S printer (PrusaResearch/Prague, Czech Republic) equipped with a nozzle with a diameter of 0.4 mm. For all samples, the nozzle temperature was 215 °C, and the bed temperature was also constant at 60 °C. The printing speed for most samples was about 50 mm/min for the infill and 40 mm/s for the outer layer (shell). All samples were prepared with the solid infill (100% infill). G-code files were generated in dedicated software Prusa Slicer Edition 2.3.3 (PrusaResearch/Czech Republic).

##### Injection

The injection molding procedure was performed using hydraulic injection molding machine model Engel ES 80/20 HLS (Engel GmbH/Schwertberg, Austria). Samples were prepared using the following parameters: injection temperature of 210 °C, injection/holding pressure of 1200/550 bar, and holding/cooling time of 10/30 s. The mold temperature was set to 25 °C, and the temperature was controlled using water cooling system.

#### 2.2.2. Sterilization

The fabricated prototypes in medical packaging system were sterilized by accelerated electrons (dose 25 kGy) at Institute of Nuclear Chemistry and Technology (Warsaw/Poland). 

#### 2.2.3. Degradation under Accelerated Conditions

The accelerated in vitro degradation process of the fabricated implant prototypes was carried out using the guides described in PN-EN ISO 10993-13:2012 “Biological evaluation of medical devices—Part 13: Identification and quantification of degradation products from polymeric medical devices” at temperature of 58 °C for 72 days. Demineralized water, hydrogen peroxide (3%; *v*/*v*), saline (0.9 wt% NaCl solution), or artificial plasma was applied as a degradation medium. The proportion of the tested sample to degradation medium was estimated based on the guide in PN-EN ISO 10993-12:2010 “Biological evaluation of medical devices—Part 12: Sample preparation and reference materials”.

The studied sample (1 g) was placed in the 10 cm^3^ of each degradation medium. At specified intervals (1, 7, 14, 21, 30, 40, 50, 61, and 72 days), samples were taken for the determination of the percentage loss of density and weight. In parallel, the determination of the pH value (CP505 pH-meter; Elmetron/Zabrze, Poland) in the degradation media was performed. 

The loss of sample density was calculated according to the following formula:

(1) $$\frac{d_{0}-d_{1}}{d_{0}}*100\%$$

where:

*d*_0_—initial density of the sample (g/cm^3^);

*d*_1_—density of the sample after each degradation interval (g/cm^3^).

The loss of the sample weight was calculated according to the following formula:

(2) $$\frac{m_{0}-m_{1}}{m_{0}}*100\%$$

where:

*m*_0_—initial weight of the sample (g);

*m*_1_—weight of the sample after each degradation interval (g).

The density of the test samples was measured using a Radwag AS 62.X2 non-automatic electronic analytical balance (Radwag/Radom, Poland), with special care, according to an in-house research procedure. The density of solids was measured by weighing the sample in air and then in a liquid of known density and temperature. Samples were placed on specially adapted pans, allowing measurement of the density of samples both less and more dense than the liquid in which the measurement takes place. These pans are perforated, which improves the accuracy of the measurement for fragile samples.

#### 2.2.4. Accelerated Degradation Assessment

Real degradation time estimation was adopted from guide described in ASTM F1980-07:2002 “Standard Guide for Accelerated Ageing of Sterile Medical Device Packages” standard.

The acceleration test describes the transformed form of the Arrhenius equation:

(3) AAF=Q10TAA−TRT10

where: 

*AAF*—accelerated degradation factor; 

*TAA*—accelerated degradation temperature (°C); 

*TRT*—real temperature of the implant application (human body temperature) (°C); 

*Q*_10_—degradation coefficient determined on the basis of kinetics of changes in the selected material property/parameter at temperature changes of 10 °C. 

To calculate the actual degradation time, the following equation was used:

(4) AAT=365 daysAAF

where: 

*AAT*—accelerated degradation time equivalent to real time (1 year mapping). 

Assuming parameter values:

- *TAA* = 58 °C;
- *TRT* = 37 °C;
- *Q*_10_ = 2.

By inserting the above values into Formulas (3) and (4), the following value ratios were received: *AAF* = 4.29 and *AAT* = 85 days.

#### 2.2.5. Differential Scanning Calorimetry

The thermal analysis of the fabricated implant prototypes was carried out using DSC 3 (Mettler Toledo/Greifensee, Switzerland). 

Three key measurement points were selected for the thermal analysis, where the beginning of the degradation process and its further progress were observed.

Hermetically sealed aluminum pans with a volume of 40 µL (Mettler Toledo/Switzerland) were used. Temperature calibration was performed using indium and n-octane. The enthalpy was calibrated using indium.

The temperature program consisted of three stages:

Segment I: first heating 25–200 °C;

Segment II: cooling 200–25 °C;

Segment III: second heating 25–500 °C.

The temperature change rate was 10 °K × min^−1^. The purge gas was nitrogen at a flow rate of 60 cm^3^ × min^−1^.

The measurement results are presented in the form of thermograms with their full characteristics. The crystallization degree of the PLDLA samples was calculated according to the formula presented in the equation below:

(5) $$X_{c}=\frac{∆H_{m}}{∆H_{m}^{0}\cdot X_{PLA}}\cdot 100\%$$

where:

*X_c_*—index of crystallinity.

Δ*H_m_*—melting enthalpy.

$∆H_{m}^{0}$—melting enthalpy of the 100% crystalline *PLA* ($∆H_{m}^{0}$ = 109 J/g). Various theoretical values of 100 % crystalline *PLA* melting enthalpy have been reported ranging from 91 to 148 J/g [19,20,21,22,23,24,25,26]. The value of 93.1 J/g was used to calculate the index of crystallinity (*X_c_*) [23,24].

*X_PLA_*—*PLA* content (*X_PLA_* = 0.9 for the 3D-printed implant samples containing 10 wt% hydroxyapatite particles; *X_PLA_* = 1 for the implant fabricated by injection sample).

Thermal analysis was carried out for samples after 14, 21, 30, 40, 50, and 72 days of degradation. 

#### 2.2.6. Scanning Electron Microscope

The surface and pore size determination were carried out using Quanta 200 (W) (FEI/Hillsboro, OR, USA) scanning electron microscope. Samples previously layered with gold were tested in high vacuum. The distribution and average pore diameter were estimated on a base of the 118 measurements of the pores.

## 3. Results and Discussion

### 3.1. Prototype of 3D-Printed Implant

#### 3.1.1. Degradation Profile Analysis

**Figure 2:**
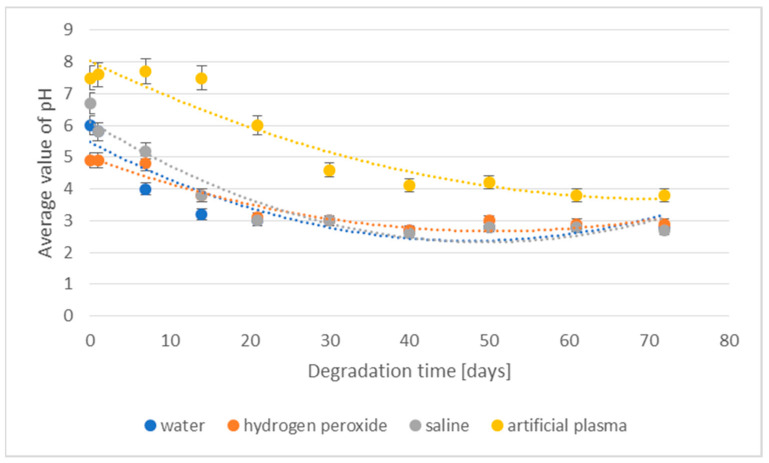
The effect of the degradation time of the prototype of the 3D-printed implant on the pH of the degradation medium.

The changes in the pH of the degradation media for the 3D-printed implant are presented in Figure 2.

In the case of samples of the 3D-printed implant placed in the media, deionized water, hydrogen peroxide, and saline, changes in pH values were noted after just 7 days of the degradation process. After 21 days, the pH stabilized and the decrease in its value was significantly lower. In the artificial plasma environment, a marked decrease in pH was noticed after only 21 days of incubation. As in the case of other media, pH stabilization for artificial plasma occurred only after 40 days of the degradation process.

**Figure 3:**
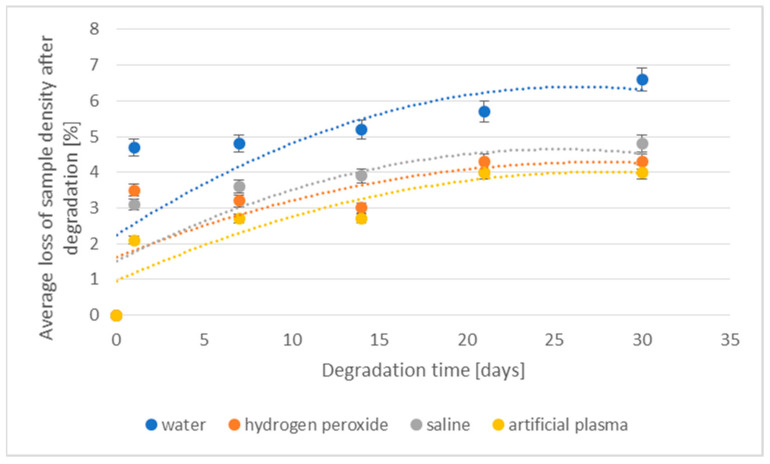
The effect of the degradation time of the prototype of the 3D-printed implant on the density loss.

**Figure 4:**
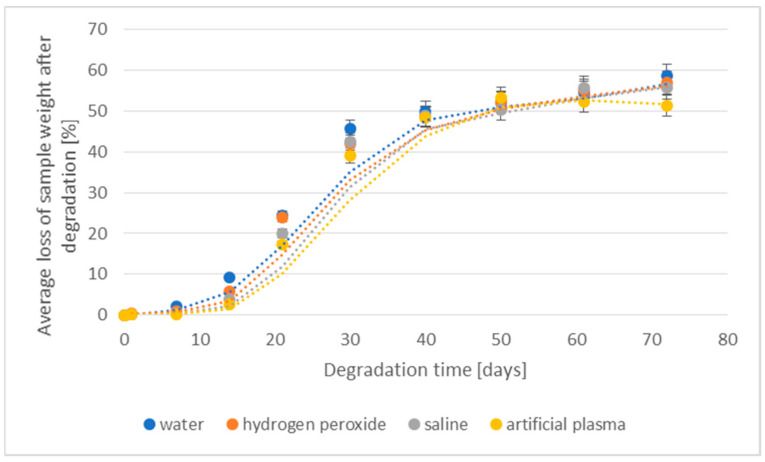
The effect of the degradation time of the prototype of the 3D-printed implant on the weight loss.

The losses of density and weight of the 3D-printed implant during the degradation in various media are shown in Figure 3 and Figure 4, respectively.

The values of the percentage loss of density of degraded samples showed a similar course regardless of the media in which they were located and a clear increase in the loss of this value was visible after 30 days of the degradation process. In the case of artificial plasma, a significant increase in the studied parameter after 21 days was observed. The largest loss of density after 1 day of degradation was observed in the case of samples placed in water (4.7%), followed by hydrogen peroxide (3.5%) and saline (3.1%). The lowest value was found for samples placed in artificial plasma (Figure 3). After 40 days of the study, it was not possible to measure the density of samples, due to the specificity of the measurement and mainly due to the progressive degradation process, causing fragility of samples.

A clear percentage loss in the weight of degradable implants was visible after 14 days of incubation (from 2.6% to 9.3%). The largest loss of weight was found for samples placed in deionized water (9.3%). For samples placed in hydrogen peroxide and saline, the loss values ranged from 3.8% to 5.7%. The lowest level of percentage loss in weight was found for an implant placed in artificial plasma. The above phenomenon is correlated with the results obtained from implant density loss studies. After 50 days, in all studied cases, a loss of more than 50% was already recorded. With the prolongation of the degradation process, the value of weight loss stabilized—after 72 days, the highest weight loss was achieved at a level of 58.7% for implants degraded in deionized water.

#### 3.1.2. DSC Results Analysis

**Figure 5:**
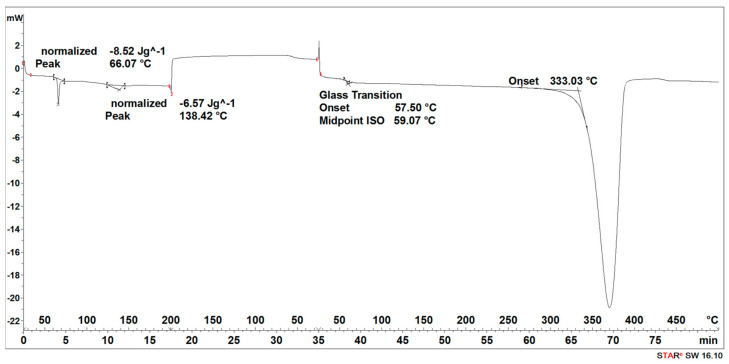
Thermal curve of initial 3D-printed implant (before acceleration degradation and after sterilization).

During the heating of the initial 3D-printed implant sample, two small endothermic peaks (SEGMENT I) were recorded, the maximum transformation temperatures of which were 63 °C and 138 °C (Figure 5).

It was observed that during the cooling of the sample (SEGMENT II), no changes were recorded in the form of peaks in the expected temperature ranges, but a slightly outlined step of about 60 °C, characteristic of the glass transition, was noticeable (Figure 5). During reheating of the sample (SEGMENT III), only a transformation around 65 °C was recorded, but no transformation around 130 °C was observed (Figure 5). The first transformation recorded on the DSC curve is related to the glass transition [27], while the second one is related to the melting point achieved through strain crystallization [28]. However, taking into account that during the cooling process of the PLDLA, only the temperature acted, without additional stress, the crystallization process did not take place. Thus, the transformation associated with the melting of the crystalline phase during re-heating (SEGMENT III) was not registered either (Figure 5). A distinct endothermic peak whose maximum transition temperature was 370 °C (SEGMENT III) is related to the thermal degradation of the sample (Figure 5).

**Figure 6:**
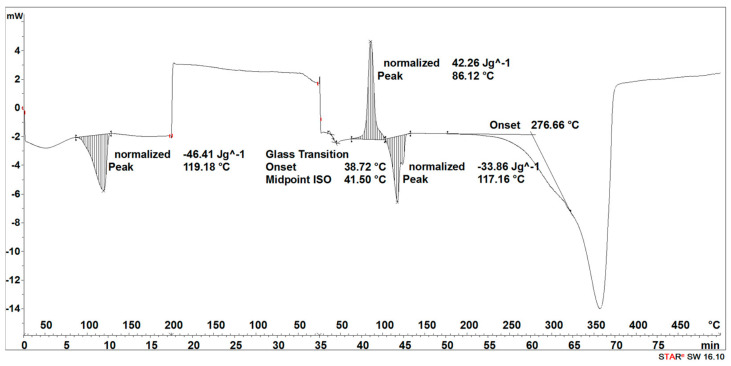
Thermal curve of 3D-printed implant after 30 days of the accelerated degradation in water.

**Figure 7:**
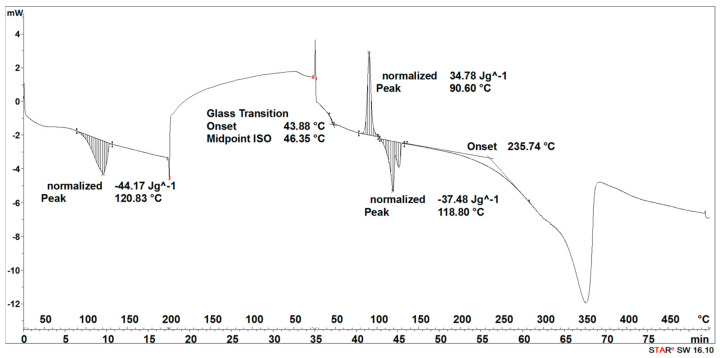
Thermal curve of 3D-printed implant after 30 days of the accelerated degradation in hydrogen peroxide.

**Figure 8:**
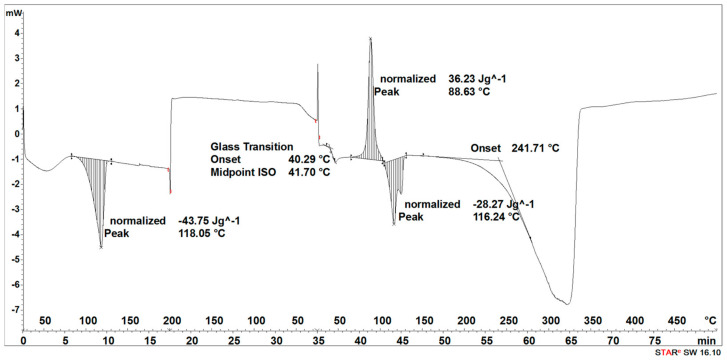
Thermal curve of 3D-printed implant after 30 days of the accelerated degradation in saline.

**Figure 9:**
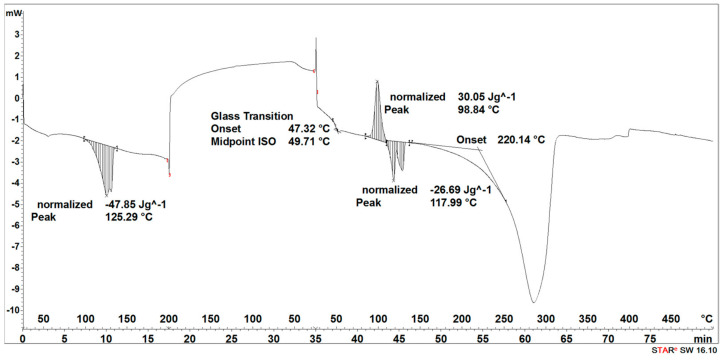
Thermal curve of 3D-printed implant after 30 days of the accelerated degradation in artificial plasma.

The degradation process of the 3D-printed implant sample (after 14, 21, 30, 40, 50, and 72 days) affected the characteristics of the thermal curves, on which the endothermic peak related to the melting of the crystalline phase—SEGMENT I was clearly outlined (Figure 5, Figure 6, Figure 7, Figure 8 and Figure 9). This was reflected in the values of the index of crystallinity (*X_c_*) (Table 1). The index of crystallinity was higher the longer the sample remained in the degradation medium. The largest increase in *X_c_* of the 3D-printed implant placed in water and in saline was observed after 50 days and achieved 61% (water) and 58% (saline), respectively (Table 1). The maximum value of *X_c_* of the 3D-printed implant placed in hydrogen peroxide was observed after 40 days and achieved 64%, while that placed in artificial plasma after 40 days achieved 62%, confirming the effect of the increase in crystallinity with the amorphous phase reduction during the hydrolytic degradation in the studied terms. 

During the cooling (SEGMENT II), the peak associated with the crystallization process was not observed. It appeared only when the sample was reheated (SEGMENT III) (Figure 6, Figure 7, Figure 8 and Figure 9). The endothermic peak from the melting of the crystalline phase recorded during the second heating (SEGMENT III) was characterized by a lower area. So, it can be assumed that during the second heating of the tested samples at constant speed, the unification of the crystalline structure of the PLDLA occurred. This was reflected in the values of the index of crystallinity after reheating (*Xc_II_*) (Table 1). This is due to the fact that the hydrolytic degradation of PLDLA takes place in the aqueous environment in which the degradation process was carried out (carbon dioxide is formed, reducing the pH—Figure 2). The hydrolytic degradation of PLDLA begins with the penetration of water into the polymer structure, which leads to the hydrolysis of ester bonds, primarily in the amorphous phase of the polymer, and the formation of shorter polymer chains, including a certain pool of oligo- and monomers [29]. The hydrolytic degradation of polylactide is accompanied by an increase in the degree of crystallinity [30,31]. 

Table 1 presents the relation of the degradation of the period and the type of degradation medium on the crystallinity index.

### 3.2. Prototype of Implant Fabricated through Injection

#### 3.2.1. Degradation Profile Analysis

**Figure 10:**
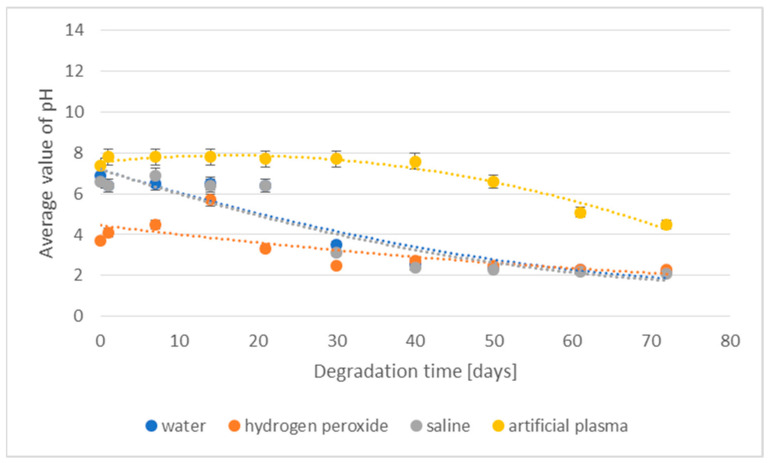
The effect of the degradation time of the prototype of the implant fabricated by injection on the pH of the degradation medium.

The effect of the degradation of the implant prototype fabricated through injection in various media on their pH is presented in Figure 10. 

In the case of a sample of the implant placed in demineralized water, no significant changes in the pH value were noted for 21 days, while after 30 days of conducting the degradation process, a clear decrease was observed, indicating a progressive degradation process. The application of 3% hydrogen peroxide solution for degradation resulted in pH stability for 14 days. The first signs of degradation were visible after 21 days. For the saline medium of degradation, for 21 days, the pH showed a quite high stability, while after 30 days, a decrease was recorded, indicating a progressive degradation process. In the artificial plasma environment, a clear decrease in pH could only be noticed after 40 days of incubation. The phenomenon of lowering the pH during the degradation in vitro for implants made using the injection technique is radically different than in the case of 3D-printed implants.

**Figure 11:**
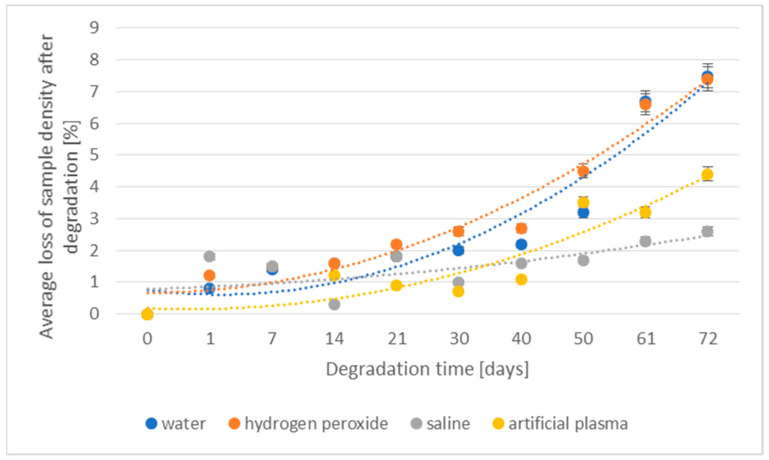
The effect of the degradation time of the prototype of the implant fabricated through injection on the density loss.

The change in the density during the degradation of the prototype of the implant made using injection in various media is shown in Figure 11.

The percentage loss of density of the degraded samples for 30 days showed a similar course regardless of the environment in which they were placed. A marked increase in density loss was visible only after 61 days of the degradation process, especially for samples placed in deionized water and hydrogen peroxide (decrease by approx. 6.7%). Until then, the loss had progressed to a small extent and at a stable level. In the case of saline and artificial plasma, significantly lower results were obtained during this degradation period (saline—2.3%, artificial plasma—4.4%).

**Figure 12:**
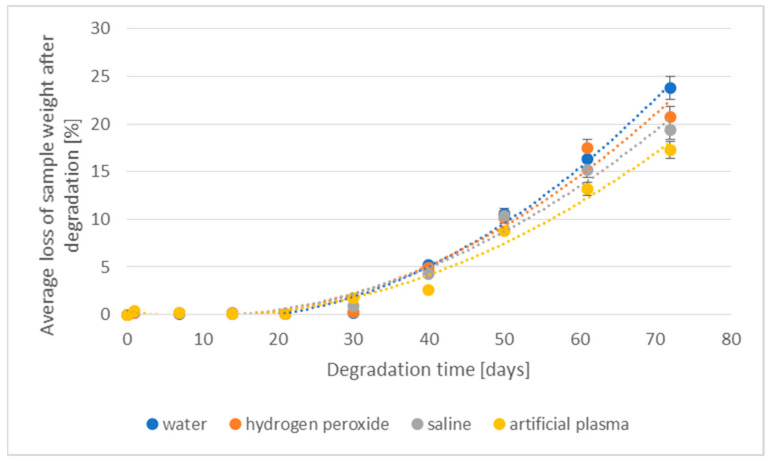
The effect of the degradation time of the prototype of the implant fabricated through injection on the weight loss.

The loss of the weight of the prototype of the implant fabricated through injection during the degradation in various media is presented in Figure 12.

Like the results of the percentage loss of weight, initially stable, the increase in weight loss occurred after 40 days, with the largest decrease recorded after 50 days of the process. The maximum value of weight decrease was obtained for samples placed in deionized water (decrease by 23.8%) and hydrogen peroxide (decrease by 20.8%), and the lowest value of this parameter for implant samples degraded in artificial plasma (only 17.3%). 

The impact of the degradation environment for implants produced through 3D printing or injection was similar, i.e., the largest changes in the test parameters were obtained for samples placed in deionized water, and the smallest for samples degraded in artificial plasma. However, the levels of these parameters and the degradation profile were significantly dependent on the origin of implant technology. Implants made using the injection technique were significantly more resistant to degradation. The weight loss was more than twice smaller than in the case of using the 3D printing technique to produce them. In addition, 3D-printed implants lost their form after 40 days of degradation and became fragile and susceptible to damage. The above observations are probably related to their internal structure and susceptibility to degradation in the entire volume of the implant. The production of a biodegradable implant using the injection technique allows a compact structure resistant to degradation factors to be obtained. 

#### 3.2.2. DSC Results Analysis

**Figure 13:**
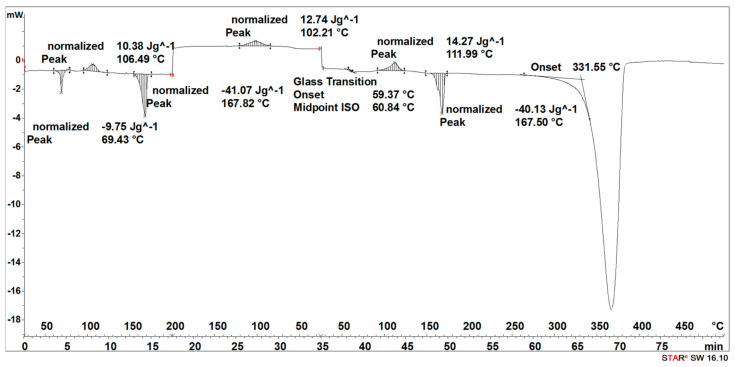
Thermal curve of initial implant fabricated through injection (before acceleration degradation and after sterilization).

During the heating of the implants fabricated by the injection sample (SEGMENT I), exothermic and two endothermic peaks were recorded (Figure 13). The first transformation around 69 °C is related to the glass transition and the melting point, the second one with the maximum at 106 °C is related to the crystallization process, and the third one at 168 °C is related to the second melting point (Figure 13).

The second stage (SEGMENT II)—the cooling process—recorded an exothermic peak connected with the crystallization process with a maximum at 102 °C and a slightly outlined step of about 60 °C, characteristic of the glass transition (Figure 13). During reheating of the sample (SEGMENT III), a glass transition at 59 °C, an exothermic peak at 112 °C, and an endothermic peak around 168 °C were recorded. A distinct endothermic peak whose maximum transition temperature was 367 °C (SEGMENT III) is related to the thermal degradation of the sample.

**Figure 14:**
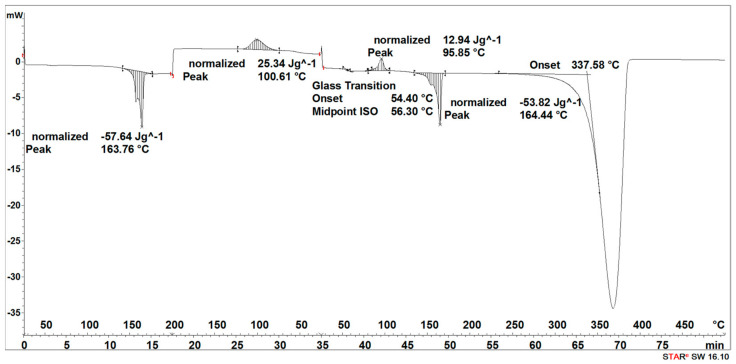
Thermal curve of implant fabricated through injection after 30 days of the accelerated degradation in deionized water.

**Figure 15:**
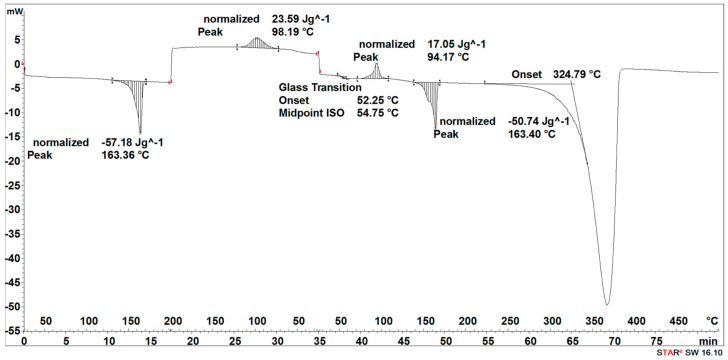
Thermal curve of implant fabricated through injection after 30 days of the accelerated degradation in hydrogen peroxide.

**Figure 16:**
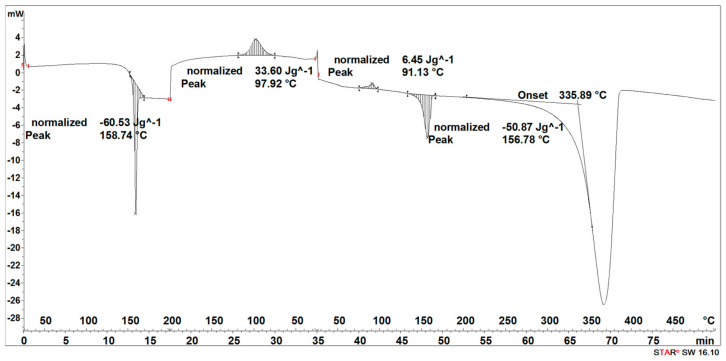
Thermal curve of implant fabricated through injection after 30 days of the accelerated degradation in saline.

**Figure 17:**
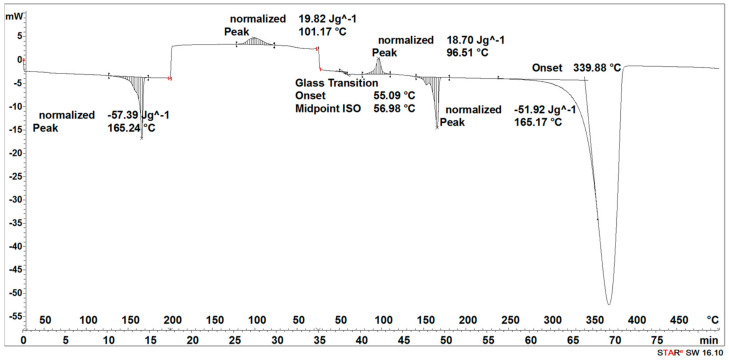
Thermal curve of implant fabricated through injection after 30 days of the accelerated degradation in artificial plasma.

The degradation process of the implant sample fabricated through injection (after 14, 21, 30, 40, 50, and 72 days) affected the characteristics of the thermal curve during the first heating (SEGMENT I), in which a single endothermic peak related to the melting of the crystalline phase was clearly outlined (Figure 14, Figure 15, Figure 16 and Figure 17). This was reflected in the values of the index of crystallinity (*X_c_*) (Table 2). The index of crystallinity was higher the longer the sample remained in the degradation medium (water, saline, and hydrogen peroxide). In that case, the increase in *X_c_* was directly proportional to the degradation time. However, there was a marked increase and then a decrease in the *X_c_* value after 30 and 40 days. The largest increase in *X_c_* of the implant sample fabricated through injection placed in water, saline, and hydrogen peroxide was observed after 72 days—near 70% (Table 2)—confirming the effect of the increase in the crystallinity with the amorphous phase reduction during the hydrolytic degradation in the studied terms. In the case of samples kept in an artificial plasma environment, the highest *X_c_* value was recorded after 14 days (69%). Continuing to hold the sample in this environment had a non-uniform effect on the *X_c_* values. It can be assumed that in the presence of artificial plasma, it does not affect the growth of crystallization to such an extent as in water, hydrogen peroxide, or saline.

It was observed that the crystallinity index after reheating *Xc_II_* of the implant fabricated through injection reached the highest values after 30–40 days of degradation in water (55% or 58%, respectively) and saline (54% or 56%, respectively) (Table 2). The maximum value of *Xc_II_* of the implant fabricated through injection placed in hydrogen peroxide was observed after 72 days and achieved 49%, while that placed in artificial plasma after 30 days achieved 56% (Table 2).

Table 2 presents the relation of the degradation of the period and the type of degradation medium on the crystallinity index. 

### 3.3. Estimation of the Real Degradation Time of Implants Depending on the Technology Used

The relation of the in vitro degradation time to the actual conditions in which the implant resides can only be approximate, due to their complexity. However, the information obtained allows us to estimate the risk of possible loss of safety and performance by the implanted medical device. 

Table 3 presents an estimate of the translation of accelerated in vitro degradation time to the time under in vivo conditions calculated according to Formulas (3) and (4). 

The tested implants were partially degraded under accelerated conditions for 72 days, which corresponded, according to the adopted assumptions and Formulas (3) and (4), to more than 10 months in the human body. Taking into account the relation of degradation time under accelerated conditions (estimated in vitro degradation temperature of 58 °C) to the real conditions of implant use (human body temperature 37 °C), it should be pointed out that the 3D-printed implant containing nanohydroxyapatite lost up to 60% of their weight (from 51.3% to 58.7% depending on the degradation medium used) during accelerated degradation corresponding to degradation under real conditions in about 10 months. In the same period of time, the implant made through injection lost only up to 24% of their weight (from 17.3% to 23.8% depending on the degradation medium used). 

Implants made through injection showed an increase in crystallinity index during the whole period of the accelerated degradation (about 23 weeks under real conditions), whereas a decrease in the discussed parameter of the 3D-printed implant after 40–50 days of the degradation (depending on the degradation medium) was observed. The above phenomenon is associated with starting the degradation of crystalline regions. The significant increase in the crystallinity index after 14 days of the accelerated degradation (related to approx. 8 weeks in real time) was observed for all media and the tested implants. The highest rise in the discussed parameter (form 44% to 69%) after 14 days for the implant made through injection and degraded in artificial plasma was found. 

Therefore, it seems—taking into account the time of bone tissue remodeling—that implants made using 3D printing technology are more biomimetic, and the process of their bone growth and superstructure will be more effective in the context of maintaining a consensus between the required mechanical strength and implant integration processes. It is estimated that the implant prototype made through injection took at least twice as long (given their degradation profile) to achieve a comparable level of weight loss.

**Figure 18:**
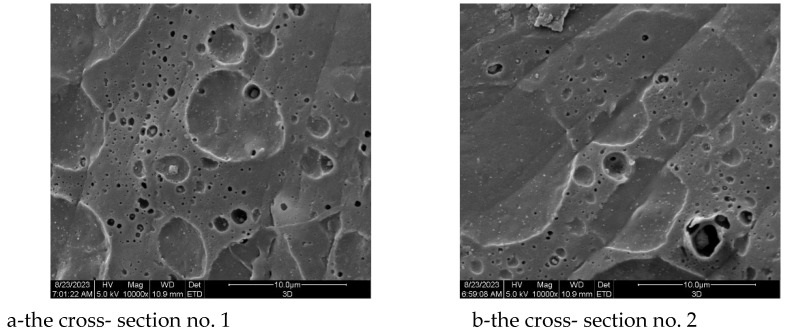
SEM microphotographs of the cross-section of the sterilized samples of implant fabricated through 3D printing (mag. ×10,000).

The presence of nanohydroxyapatite promotes the degradation process in the case of implant production using the 3D printing technique. First, the 3D printing technique enables a controlled process of forming pores inside the implant constituting hydrolytic degradation foci inside the implant, which accelerates the process of degradation itself. The two-point cross-section through the 3D sample are presented in Figure 18a,b. 

**Figure 19:**
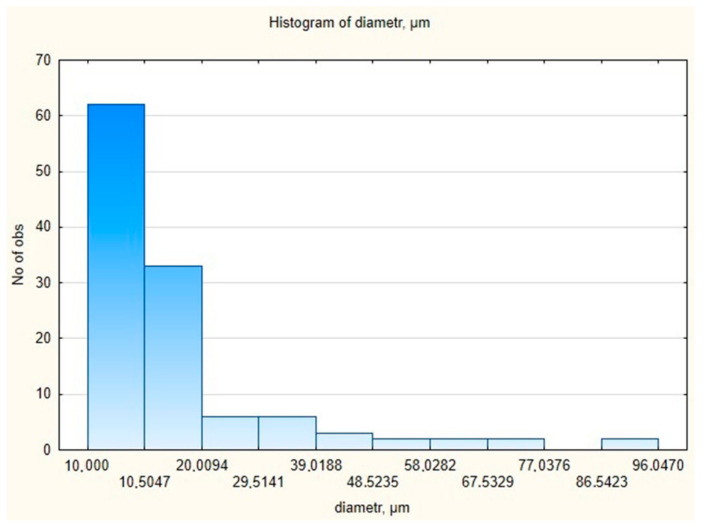
The histogram of the pore diameter distribution in 3D-printed implant.

The cross-section of the 3D-printed implants is characterized by the presence of a system of pores with various sizes, and with an average diameter of 16.10 μm and main diameter distribution range from 1 to 96.05 μm. The histogram of the pore diameter distribution in the prototype of the 3D-printed implant is shown in Figure 19.

**Figure 20:**
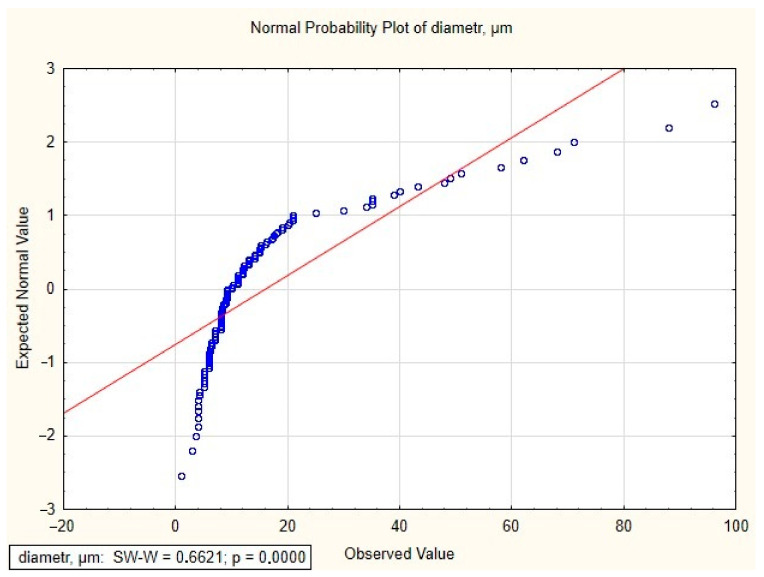
The normal probability plot of pore diameter in 3D-printed implant.

The relative standard deviation (RSD) was 105.9%, which indicates a large distribution of pore diameters. However, the median range was 9.61, indicating the value most frequently occurring in the samples and a right-sided inclined distribution. The distribution of pore diameters indicated the presence of more numerous lower values of the determined parameter. An analysis was also carried out to determine whether the pore diameter distribution had a normal distribution using Statistica 12.5.192.7 software. The Shapiro–Wilk test was used. Pore diameter is not a random variable with a normal distribution, because the *p*-value is less than α = 0.05 (Figure 20).

**Figure 21:**
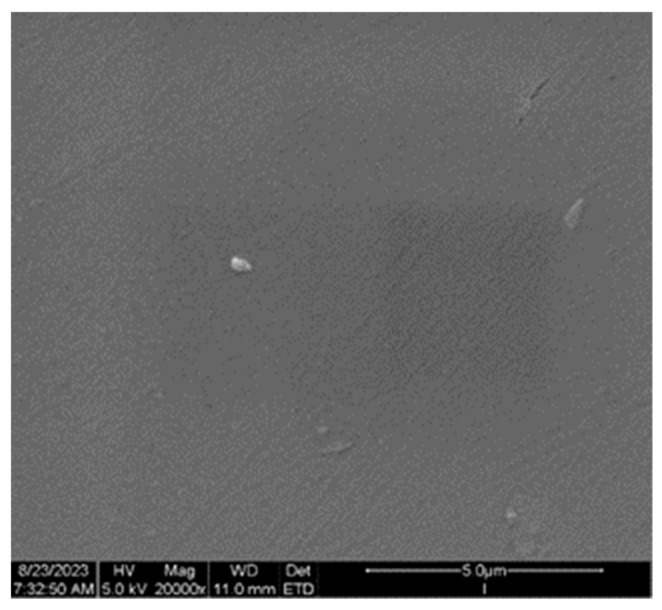
SEM microphotograph of the cross-section of the sterilized samples of implant fabricated through injection (mag. × 20,000).

The above-discussed pores promote the enhancement of the surface contact with degradation media and support the quicker degradation in vitro. Secondly, the introduction of nanohydroxyapatite particles enables the induction of the bone growth process and the strengthening of the biocomposite. The cross-section of implant samples made through injection was characterized by a flat surface without visible pores, which results directly from the technology used (Figure 21).

## 4. Conclusions

The method of fabrication and composition of the biodegradable implant obviously shape its susceptibility to degradation processes in vitro. Implants manufactured through injection were more resistant to degradation in vitro, which was reflected in a continuous increase in the crystallinity index, while 3D-printed implants were distinguished by faster weight loss with a decrease in crystallinity after at least 40 days of degradation.

When selecting implant technologies, the intended use and the expected minimum degradation time in the body should be taken into account, especially in the case of biodegradable implants, to ensure the possibility of bone tissue restoration without the risk of loss of physical integrity of the implant and the minimum required strength.

The structure of the 3D-printed implant supports the degradation process under accelerated conditions, especially inside the implant, which has been documented especially in weight loss and crystallinity change studies.

Moreover, the aspect of the possibility of controlling the degradation process through technology parameters, structure, and composition is very interesting due to the personalization of the implants in terms of the aimed clinical localization, surgical requirements, and recommendation as well as age of the patient.

It is obvious that the research presented in this paper will be validated under in vivo conditions, which will allow the determination of the comparability of the obtained results, taking into account the aspect of the complexity of the degradation process in the body. Moreover, the aspects of micro- and macroanalysis of fabricated implant changes during the degradation in vitro process, as well as the composition of the post-degradation mixture due to the volume of the research material, will be the subject of the next publication.

## Figures and Tables

**Table 1:** Effect of the degradation period of the 3D-printed implant on the index of crystallinity of the 3D-printed implant after first heating (*X_c_*) and after reheating (*Xc_II_*).

Degradation Period	*Xc* (%)	*Xc_II_* (%)
Prototype of the 3D-printed implant before degradation (sterilized)	8	-
degradation medium: deionized water		
14 days	33	32
21 days	55	44
30 days	55	40
40 days	57	48
50 days	61	30
72 days	45	17
degradation medium: hydrogen peroxide		
14 days	27	27
21 days	35	32
30 days	53	45
40 days	64	37
50 days	51	32
72 days	51	15
degradation medium: saline		
14 days	26	24
21 days	37	31
30 days	52	34
40 days	58	33
50 days	58	25
72 days	44	54
degradation medium: artificial plasma		
14 days	27	21
21 days	38	30
30 days	57	32
40 days	62	31
50 days	55	15
72 days	61	3

**Table 2:** Effect of the degradation period of the implant fabricated through injection on the index of crystallinity of the implant after first heating (*X_c_*) and after reheating (*Xc_II_*).

Degradation Period	*X_c_* (%)	*Xc_II_* (%)
Prototype of the implant fabricated through injection (sterilized)	44	43
degradation medium: deionized water		
14 days	50	43
21 days	50	45
30 days	62	58
40 days	53	55
50 days	65	52
72 days	67	57
degradation medium: hydrogen peroxide		
14 days	48	42
21 days	52	48
30 days	61	55
40 days	55	54
50 days	67	53
72 days	68	58
degradation medium: saline		
14 days	48	44
21 days	49	47
30 days	65	54
40 days	57	56
50 days	67	56
72 days	69	53
degradation medium: artificial plasma		
14 days	69	49
21 days	51	47
30 days	62	56
40 days	53	49
50 days	68	54
72 days	63	44

**Table 3:** The relation between the accelerated degradation period and the degradation of the implants under real conditions in human body.

Days of the Accelerated In Vitro Degradation	1	7	14	21	30	40	61	72
Weeks under real conditions corresponding to the accelerated in vitro degradation	0.6	3.9	7.9	11.8	16.9	22.6	34.4	40.6

## Data Availability

Not applicable.
